# New Insights into the Role of Synovial Fibroblasts Leading to Joint Destruction in Rheumatoid Arthritis

**DOI:** 10.3390/ijms24065173

**Published:** 2023-03-08

**Authors:** Kotaro Matsuda, Naoto Shiba, Koji Hiraoka

**Affiliations:** Department of Orthopedic Surgery, Kurume University School of Medicine, 67 Asahi-machi, Kurume 830-0011, Fukuoka, Japan

**Keywords:** rheumatoid arthritis, synovial fibroblast, RANKL, joint destruction, single cell analysis

## Abstract

Rheumatoid arthritis (RA), one of the most common autoimmune diseases, is characterized by multiple-joint synovitis with subsequent destruction of bone and cartilage. The excessive autoimmune responses cause an imbalance in bone metabolism, promoting bone resorption and inhibiting bone formation. Preliminary studies have revealed that receptor activator of NF-κB ligand (RANKL)-mediated osteoclast induction is an important component of bone destruction in RA. Synovial fibroblasts are the crucial producers of RANKL in the RA synovium; novel analytical techniques, primarily, single-cell RNA sequencing, have confirmed that synovial fibroblasts include heterogeneous subsets of both pro-inflammatory and tissue-destructive cell types. The heterogeneity of immune cells in the RA synovium and the interaction of synovial fibroblasts with immune cells have recently received considerable attention. The current review focused on the latest findings regarding the crosstalk between synovial fibroblasts and immune cells, and the pivotal role played by synovial fibroblasts in joint destruction in RA.

## 1. Introduction

Rheumatoid arthritis (RA) is the most frequent autoimmune disease and is characterized by multiple-joint synovitis, leading to joint destruction throughout the body. The patient subpopulation is etiologically heterogeneous, with a complex interaction of genetic background with environmental causes, such as smoking, periodontal disease, and microbial flora [1,2]. The appearance of autoantibodies, namely rheumatoid factor (RF) and/or anti-citrullinated peptide antibodies (ACPAs), is a hallmark of RA, and simultaneously, a criterion for diagnosis and prediction of disease prognosis. However, some patients are seronegative, suggesting that innate immune disorders are also involved in the pathogenesis of RA [3,4].

RA is attributed to the interaction of pathological T cells and synovial cells, which promotes the expression of receptor activator of NF-κB ligand (RANKL) on synovial fibroblasts resulting in osteoclast differentiation, and then, bone resorption [5,6,7]. The first step is the pro-inflammatory cytokine production by activated lymphocytes, macrophages, and neutrophils, which stimulates synovial fibroblasts. Synovial fibroblasts differentiate into pro-inflammatory and tissue-destructive subtypes [8,9]. Tissue-destructive synovial fibroblasts express RANKL and induce osteoclast differentiation causing bone destruction; they additionally produce tissue-degenerative enzymes, such as matrix metalloproteinases (MMPs), that promote cartilage destruction [10]. As understanding of the pathogenesis of the disease has advanced, antibody drugs targeting tumor necrosis factor (TNF) [11], interleukin-6 (IL-6) [12], and RANKL [13] have been clinically applied in addition to conventional methotrexate (MTX). Moreover, novel molecules, such as Janus kinase (JAK), have become drug targets, resulting in major progress in treatment [14,15]. The drugs have not only improved the course of RA drastically but also significantly contributed to our comprehension of RA [16].

However, current therapy is not effective for all patients; there are cases of “difficult-to-treat (D2T) RA” that never reach remission [17]. In addition, these therapies can elicit numerous side effects, such as infusion reactions and serious infections. Therefore, the identification of pathogenic cell subsets and pathogenic molecules specific to affected joints, and the development of novel therapies targeting them, are urgently required.

In RA, activated autoimmunity causes bone destruction. Many research groups have shown that RANKL is highly expressed in the inflamed synovium and induces bone destruction. Recently, state-of-the-art analytical technologies, such as single-cell RNA sequencing (scRNA-seq), have shed light on various pathogenic synovial cell subsets of RA, including synovial fibroblasts [8,9,10,18,19,20]. In this review, we discuss the interplay of synovial fibroblasts with immune cells and suggest new perspectives on the properties and heterogeneity of synovial fibroblasts that cause structural destruction in RA.

## 2. Mechanisms of Immune Dysregulation in RA

**Figure 1:**
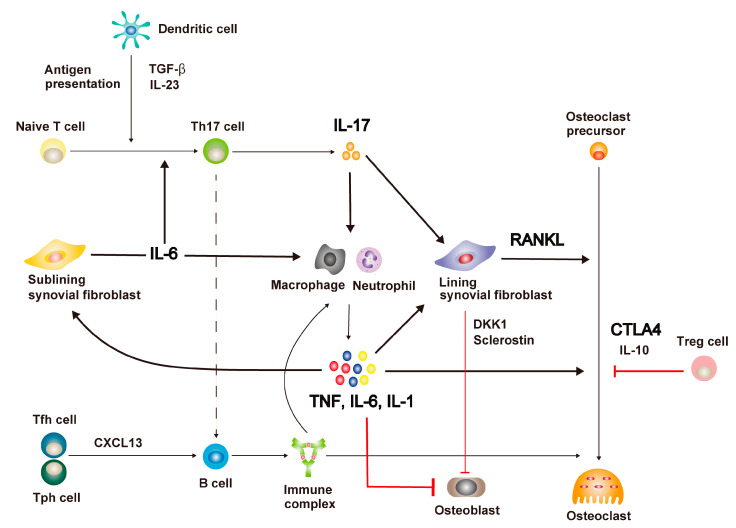
The pathway of inflammation and bone destruction in rheumatoid arthritis and the mechanism of action of biological agents. In rheumatoid arthritis (RA) synovium, antigen-presenting cells (APCs), including dendritic cells, present self-antigens and produce transforming growth factor-β (TGF-β) and interleukin-23 (IL-23), which promote T helper 17 cell (Th17) differentiation and IL-17 secretion. IL-17 stimulates the production of pro-inflammatory cytokines, such as tumor necrosis factor (TNF), IL-6, and IL-1, by synovial macrophages and neutrophils, and induces receptor activator of NF-κB ligand (RANKL) expression in lining synovial fibroblasts. Pro-inflammatory cytokines further promote IL-6 production by the sub-lining synovial fibroblasts and RANKL expression in the lining of synovial fibroblasts and activate the osteoclast differentiation pathway. Immune complexes produced by B cells, upon stimulation of chemokine (C-X-C motif) ligand 13 (CXCL13) from T follicular helper (Tfh) and T peripheral helper (Tph) cells, boost local inflammation and osteoclast differentiation. Dickkopf-related protein 1 (DKK1) and sclerostin, produced by the lining synovial fibroblasts, and pro-inflammatory cytokines inhibit osteoblast differentiation. Anti-IL-17 antibodies suppress inflammation and bone destruction. Anti-TNF antibodies and anti-IL-1 antibodies inhibit inflammation and bone destruction, simultaneously improving bone repair capacity. Anti-IL-6 antibodies restrain inflammation, bone destruction, and Th17 cell differentiation besides contributing to the improvement in bone repair capability. CTLA4 immunoglobulin prevents osteoclast differentiation. Anti-RANKL antibody directly inhibits bone destruction.

Excessive autoimmune responses underlie the pathogenesis of RA. Activated immune cells trigger synovial fibroblasts, which are normally structural mesenchymal cells of joints, to produce pro-inflammatory cytokines and tissue-degenerative proteins, including RANKL, leading to the inflammation and joint destruction seen in RA. Recent advances in analytical methods have focused on the heterogeneous immune cell subsets of RA synovium. The activation of the immune system leading to impaired bone metabolism and the therapeutic target molecules of RA are discussed in this section (Figure 1).

### 2.1. Immune Cell Activation

CD4+ naïve T cells that recognize self-antigens presented by antigen-presenting cells (APCs), including dendritic cells, differentiate to T helper 17 (Th17) cells upon stimulation by transforming growth factor-β (TGF-β), IL-23, and IL-6. Th17 cells infiltrate into the RA synovium and secrete IL-17, the crucial cytokine for autoimmune inflammation. IL-17 activates innate immune cells, such as synovial macrophages and neutrophils, to induce local inflammation, resulting in the production of pro-inflammatory cytokines, including TNF, IL-6, and IL-1 [21,22]. Pro-inflammatory cytokines promote IL-6 production by sub-lining synovial fibroblasts and activate the osteoclast differentiation pathway. T follicular helper (Tfh) cells, present in the draining lymph nodes, and T peripheral helper (Tph) cells, the newly identified T cell subset present in inflamed synovium, secrete chemokine (C-X-C motif) ligand 13 (CXCL13) to stimulate B cells producing autoantibodies, such as RF and ACPAs, which then form immune complexes [23]. Immune complexes further activate pro-inflammatory cytokines and chemokines that promote osteoclast differentiation [24]. In addition, Th17 cells promote the production of desialylated immunoglobulin G, which increase inflammation, by inhibiting the expression of *St6gal1* in B cells via IL-21 and IL-22 [25]. Lining synovial fibroblasts, expressing RANKL in response to the stimulation by IL-17 and other pro-inflammatory cytokines, strongly induce osteoclast differentiation [21,22]. Additionally, synovial fibroblasts stimulated by TNF produce Dickkopf-related protein 1 (DKK1) and sclerostin to inhibit osteoblast differentiation [26,27]. Therefore, bone repair fails to function properly, in addition to bone destruction, resulting in a state of impaired bone metabolism in RA.

### 2.2. Representative Therapeutic Target Molecules

Representative therapeutic target molecules in RA include TNF, IL-6, IL-1, CTLA4, IL-17, RANKL, and JAKs [11,12,13,22,28,29,30].

TNF is mainly produced by macrophages, although T cells and B cells are also sources of production. TNF activates immune cells and promotes bone resorption by increasing RANKL expression in lining synovial fibroblasts [18,21,22]. In addition, TNF plays a role in inhibiting bone formation by suppressing osteoblast differentiation [31]. Anti-TNF antibodies can be effective against bone destruction by suppressing RANKL as well as inflammation [21,22]. Furthermore, anti-TNF antibody had previously been reported to suppress bone destruction even when it could not suppress inflammation [32], suggesting that TNF regulation is important for improving bone metabolism.

IL-6 is produced primarily by the sub-lining synovial fibroblasts [18,21,22]. It plays a central role in the pathophysiology of RA by enhancing local inflammation, stimulating the differentiation of Th17 cells, and inducing RANKL expression in the lining synovial fibroblasts, thereby promoting osteoclast differentiation [21,22,33]. Thus, anti-IL6 receptor antibodies not only inhibit inflammation and bone destruction but also have therapeutic effects against Th17 cell differentiation.

IL-1, produced chiefly by macrophages, induces local inflammation and RANKL production by synovial fibroblasts and suppresses osteoblast differentiation [18,21,22,34].

The main source of CTLA4 is the regulatory T (Treg) cell [35]. Abatacept (CTLA4-immunoglobulin (Ig)) prevents the binding of CD80/CD86 in dendritic cells to CD28 in T cells and reduces T cell activity, thereby resolving inflammation [36]. Moreover, it suppresses osteoclast differentiation by promoting apoptosis of osteoclast progenitor cells, and the underlying mechanism is independent of CD80/CD86 [37].

Th17 cells play a very important role in autoimmune arthritis, producing IL-17. The latter activates synovial macrophages and promotes RANKL expression in the lining of synovial fibroblasts [38,39]. In addition, Th17 cells enhance the pro-inflammatory effects of autoantibodies through the desialylation of autoantibodies [25]. Although findings from studies on various murine models of arthritis have suggested IL-17A to be essential for both inflammation and bone destruction, inhibitors of IL-17A did not have sufficient effect compared to other biological agents [40]. The heterogeneity of patients with RA is one of the causes of the ineffectiveness of IL-17A inhibitors. However, favorable results could be achieved in patients with RA who did not respond to a TNF inhibitor by dual blockage of IL-17A and IL-17F, which indicated that blockage of IL-17 could be a promising option for some patients with RA [41].

RANKL, produced by tissue-destructive synovial fibroblasts, is a crucial cytokine for osteoclastogenesis and a major player in bone destruction in RA. The discovery of RANKL was the cornerstone in the study of bone destruction in RA [21,42]. In Japan, the anti-RANKL antibody denosumab is used in daily clinical practice for the treatment of RA-related bone destruction, osteoporosis, and bone metastasis of malignant tumors [13,43].

JAKs are expressed by different kinds of cells in the inflamed synovium. JAKs phosphorylate signal transducers and activators of transcription proteins (STATs), which then regulate gene transcription in the nucleus [44]. Although most immune cells are affected by the JAK-STAT pathway, it is unclear which types of cells and signaling pathways are actually specific therapeutic targets. JAK inhibitors have been reported to reduce interferon-γ (IFN-γ) and IL-17 production by T cells [45] and suppress CD80/CD86 along with TNF and IL-6 from dendritic cells [46]. In addition, the effect of JAK inhibitors in improving bone metabolism in RA has attracted much attention. Although JAK inhibitors do not have a direct influence on osteoclast precursor cells, they suppress RANKL expression in mesenchymal osteoclast differentiation-supporting cells [47,48]. In contrast, JAK inhibitors have been reported to activate the Wnt pathway to promote osteoblast differentiation in vitro [47]. These findings suggested that JAK inhibitors not only prevent bone destruction but also promote bone formation under inflammatory conditions. Therefore, further studies, especially in vivo, are warranted.

## 3. Immune Cell-Mediated Bone Destruction in RA

In RA, bone resorption by osteoclasts is excessively accelerated, whereas bone formation by osteoblasts is suppressed. Our current understanding of the pathogenesis of bone destruction in RA has evolved from the studies on RANKL [21,49]. Although bone erosion has been extensively studied in RA, recent years have seen a growing interest in periarticular bone loss and systemic bone loss. In this section, we aim to provide a comprehensive review of historical and recent trends in studies on bone destruction in RA, including periarticular bone loss and systemic bone loss.

### 3.1. Excessive Bone Resorption in RA as RANKL Disease

The reason underlying bone resorption due to autoimmune disease has long been unclear. Some researchers considered that proteolytic enzymes produced by inflammation might destroy bones [50]; however, osteoclasts were recognized to be essential for rapid bone resorption in the area of bone metabolism. In fact, a large number of osteoclasts were observed at the interface, where the synovium invaded the bone, in the pathological analysis of RA synovium [51]. Moreover, osteoclasts were found to be generated by culturing synovial tissue from patients with RA, thereby indicating the presence of both osteoclast precursor cells and osteoclastogenesis-supporting cells in RA synovium [52]. Subsequently, RANKL was discovered and noted to be highly expressed in RA synovium [42,53]. Mice lacking osteoclasts were found to not experience bone destruction despite the induction of arthritis [54]. From a therapeutic perspective, bone destruction was reported to be prevented by suppressing osteoclasts and RANKL expression [5,55]. Thus, the concept that bone destruction in RA is a pathological condition in which RANKL is abnormally expressed was established.

### 3.2. The Main Source of RANKL Is Synovial Fibroblast

Despite the aforementioned information, there is no unified view of the source of RANKL in RA. While T cells infiltrating the inflamed synovium have been reported to be the major cause of RANKL [5], conventionally activated T cells have been shown to suppress RANKL by producing IFN-γ [56]. At that time, immune pathology was explained by the Th1/Th2 paradigm, and RA was considered to be a Th1-type disease. However, as mentioned above, since IFN-γ suppressed osteoclast differentiation, it is unlikely that bone destruction in RA is caused by Th1 cells. Subsequently, the pathological T cells with the ability to induce osteoclast differentiation were confirmed to be Th17 cells [39]. IL-17 produced by Th17 cells promotes RANKL expression in osteoclastogenesis-supporting cells, in addition to inducing TNF and IL-6, by causing local inflammation. In addition, Th17 cells themselves were found to express RANKL [39]. Nevertheless, synovial fibroblasts were speculated to possibly be the most important source of RANKL in RA, since mesenchymal cell mediation is required for osteoclast induction by Th17 cells. This hypothesis was ultimately confirmed to be correct since synovial fibroblast-specific RANKL-deficient mice did not experience bone destruction even under arthritic conditions, although the bones of T-cell-specific or B-cell-specific RANKL-deficient mice were destroyed [7]. Recent studies using scRNA-seq analysis have shown that synovial fibroblasts, especially the tissue-destructive subset, are the major source of RANKL in RA synovium [8,9,10,18].

### 3.3. RANKL Is the Master Regulator of Osteoclast Differentiation

**Figure 2:**
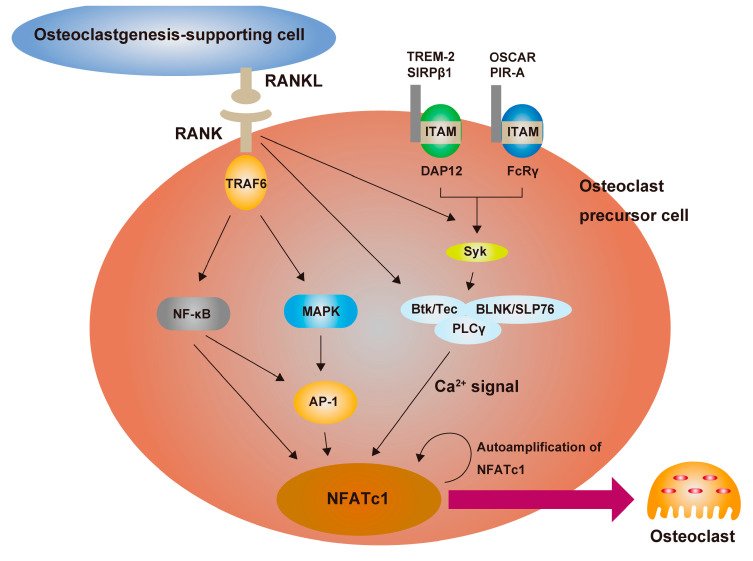
RANKL signal transduction in osteoclastogenesis. Osteoclastogenesis-supporting cells, such as osteoblasts, osteocytes, and synovial fibroblasts, express receptor activator of NF-κB ligand (RANKL) and bind to receptor activator of NF-κB (RANK) on osteoclast precursor cells. RANKL-RANK binding recruits tumor necrosis factor receptor-associated factor 6 (TRAF6), which activates NF-κB and mitogen-activated protein kinase (MAPK) signaling pathways, leading to activation of the downstream factors such as activator protein 1 (AP-1). Immunoglobulin-like receptors such as triggering receptor expressed on myeloid cells 2 (TREM-2), signal-regulatory protein β1 (SIRPβ1), osteoclast-associated receptor (OSCAR), and paired immunoglobulin-like receptor A (PIR-A), which associate with DNAX activating protein of 12 KDa (DAP12) or Fc receptor γ-chain (FcRγ), are essential molecules in transmitting co-stimulatory signals required for osteoclast differentiation. Immunoreceptor tyrosine-based activation motifs (ITAMs) are phosphorylated by both RANKL signal and immunoglobulin-like receptors signal, leading to activation of Ca2+ signaling through spleen tyrosine kinase (Syk) and complexes involving Tec kinases Btk/Tec, adaptor proteins such as B cell linker protein (BLINK)/Src homology 2 domain-containing leukocyte protein of 76 kDa (SLP76), and phospholipase Cγ (PLCγ). Ca2+ signaling induces activation and auto-amplification of nuclear factor of activated T cells 1 (NFATc1), ultimately determining osteoclast differentiation.

Osteoclasts are cells that have multiple nuclei and are derived from monocyte/macrophage progenitor cells [57]. They resorb bone matrix by producing acid and proteolytic enzymes. The differentiation of osteoclasts is dependent on signals from mesenchymal supporting cells [58]. In 1998, RANKL, a cytokine that activates dendritic cells expressed on T cells [59], was identified as being indispensable for osteoclast differentiation [60]. It was also found that osteoprotegerin (OPG) functions as a decoy receptor for RANKL, inhibiting osteoclast differentiation [61]. RANKL has since been shown to be essential for both the bone and immune systems, as evidenced by severe osteopetrosis and lymph node dysplasia observed in RANKL knockout mice [62]. RANKL binds to receptor activator of NF-κB (RANK) on osteoclast precursor cells and activates mitogen-activated protein kinase (MAPK) and NF-κB pathways through the involvement of tumor necrosis factor receptor-associated factor 6 (TRAF6) and transforming growth factor β-activated kinase 1 (TAK1) [49,58,63]. In addition, RANK cooperates with immunoglobulin-like co-stimulatory receptors such as triggering receptor expressed on myeloid cells 2 (TREM-2), signal-regulatory protein β1 (SIRPβ1), osteoclast-associated receptor (OSCAR), and paired immunoglobulin-like receptor A (PIR-A) [49,58,63]. Co-stimulatory receptors bind to adapter molecules that contain immunoreceptor tyrosine-based activation motifs (ITAMs), such as DNAX activating protein of 12 kDa (DAP12) and Fc receptor γ-chain (FcRγ) [24,64]. The phosphorylation of ITAM recruits spleen tyrosine kinase (Syk), which activates Tec kinases Btk/Tec and adaptor proteins such as B cell linker protein (BLINK) and Src homology 2 domain-containing leukocyte protein of 76 kDa (SLP76), leading to the promotion of phospholipase Cγ (PLCγ)-mediated Ca2+ signaling [65]. These intracellular signaling pathways ultimately induce and activate nuclear factor of activated T cells 1 (NFATc1). NFATc1 is a crucial molecule in the terminal differentiation of osteoclasts [66]. It induces the expression of a number of genes associated with osteoclast differentiation and also forms a positive auto-amplification loop (Figure 2).

### 3.4. Defective Bone Formation by Osteoblast in RA

In RA, bone formation is inhibited while bone resorption is accelerated. In particular, osteoblast function is impaired by pro-inflammatory cytokines in the vicinity of inflamed joints [67]. As described previously, TNF inhibits osteoblast differentiation by suppressing RUNX2 [31]. In addition, TNF regulates inhibitors of the Wnt pathway, including DKK1 and sclerostin [68]. DKK1 and sclerostin are known to block the Wnt pathway by binding to the canonical Wnt receptor and lipoprotein receptor-related protein-5 and -6 (LPR5/6). DKK1 is regulated by TNF in the inflamed synovium, and administration of anti-DKK1 antibody to arthritic mice not only inhibits bone destruction but also promotes bone formation [26]. Additionally, sclerostin is produced from synovial fibroblasts activated by TNF in the inflamed synovium as well as in osteocytes [27]. Among other typical pro-inflammatory cytokines, IL-1 inhibits osteoblast differentiation [34]. As for IL-6 and IL-17, it is controversial; IL-6 has been reported to promote osteoblast differentiation and increase bone mass [69], whereas administration of anti-IL-6 receptor antibodies in an inflammatory environment does not decrease bone mass [70]. In spondyloarthritis, IL-17 is highly influential; inflammation promotes bone formation, whereas, the inhibition of IL-17 suppresses both, bone formation as well as inflammation [71,72]. In addition, IL-17 produced by γδ T cells at a fracture site is known to facilitate bone formation [73]. In contrast, bone formation is enhanced in IL-17-deficient mice under arthritic conditions [74]. The effects of IL-6 and IL-17 on bone formation may be strongly influenced by the cells that produce them and the surrounding microenvironment. Understanding the osteoblast function in an inflammatory milieu can lead to novel therapies aimed not only at reducing bone destruction but at repairing bone metabolism.

### 3.5. Periarticular and Systemic Bone Loss

Periarticular bone loss is a condition that occurs in the bone adjacent to inflamed joints and is recognized early in the onset of RA [75]. Although the precise mechanism remains unclear, it has been reported to be present prior to the onset of arthritis in ACPA+ individuals [76]. The role of B cells has been implicated in periarticular bone loss. It was demonstrated that ACPAs induced periarticular bone loss in a mouse model of arthritis [77]. Additionally, the function of plasma cells in periarticular bone loss has recently been demonstrated. RANKL was found to be highly expressed by synovial fibroblasts in the joints, as well as by plasma cells in the bone marrow proximal to the inflamed joints in murine arthritis. Furthermore, RANKL-expressing plasma cells had an osteoclast-inducing ability. Moreover, periarticular bone loss was reduced in mice lacking RANKL in the B cell lineage, although bone erosion of the articular surfaces was not affected [78]. These findings suggest that plasma cells not only produce pro-inflammatory cytokines and autoantibodies but also express RANKL in the vicinity of inflamed joints, leading to periarticular bone loss. Another report focused on the interaction between osteoblasts and B cells under arthritic conditions. B cells in the bone marrow adjacent to inflamed joints accumulate in close proximity to osteoblasts and inhibit osteoblastogenesis by expressing chemokine (C-C motif) ligand 3 (CCL3) and TNF [79]. This suggests that osteoblasts are also regulated by the B cell lineage of the inflammatory milieu. The relationship between osteoblasts and osteocytes, which are predicted to express RANKL in the vicinity of inflamed joints, and periarticular bone loss is currently unclear, and further studies are, therefore, needed.

Systemic bone loss in RA manifests as extensive osteoporosis [68,80]. The condition arises from various factors, including aging, menopause, vitamin D deficiency, and lack of exercise, which typically result in reduced bone mineral density in the vertebrae and femurs, leading to fragility fractures [22]. RA patients have approximately double the risk of developing osteoporosis compared to the general population, indicating the existence of RA-specific risk factors [81]. Pro-inflammatory cytokines promote osteoclast differentiation and inhibit osteoblast differentiation [80], and immune complexes circulating in the body facilitate osteoclastogenesis [24,82]. Additionally, glucocorticoid treatment and impaired activities of daily living (ADL) due to joint deformities are risk factors for osteoporosis [80]. While some studies have suggested that suppression of inflammation by glucocorticoids may prevent osteoporosis [83,84], they generally hinder osteoblast differentiation and induce apoptosis, while activating osteoclasts [80]. In RA patients, reduced ADL and mechanical loading promote RANKL and sclerostin expression in osteocytes, which enhances bone resorption by osteoclasts, further exacerbating systemic bone loss [85,86]. Furthermore, many RA patients are elderly women, and menopause leads to a decrease in estrogen secretion, which has an inhibitory effect on osteoclastogenesis [87]. In addition to pharmacotherapy and surgery, prevention of decline in ADL through rehabilitation is recommended to address systemic bone loss in RA.

## 4. Mechanisms of Cartilage Degeneration in RA

Destruction of cartilage and bone is an important factor for structural destruction in RA. The mechanism of cartilage destruction may be independent of RANKL since RANKL inhibition does not prevent cartilage destruction unlike bone destruction [13].

The representative degenerative disease of cartilage is osteoarthritis (OA), which is common in elderly people. The onset of OA is affected by genetic background, as well as by environmental factors, such as excessive mechanical loading, trauma, inflammation, and various other stresses [88]. Chondrocyte phenotypic stability is impaired and extracellular matrix (ECM) degenerates in OA. The distinctive OA phenotype includes decreased SRY-box9 (Sox9) expression and increased Runx2 expression in chondrocytes, presenting a mechanism similar to endochondral ossification [88,89].

Cartilage degeneration in RA is caused by an excessive autoimmune response. Pro-inflammatory cytokines, such as TNF, IL-6, IL-1, and IL-17, stimulate the production of cartilage-destructive enzymes, including MMPs and aggrecanases (ADAMTS-4 and ADAMTS-5), by synovial fibroblasts and inhibit the production of ECM by chondrocytes [88,90]. In particular, MMP-1, MMP-3, MMP-9, MMP-13, and MMP-14 (MT1-MMP) are overexpressed by synovial fibroblasts in RA. MMP-14 is upregulated in the synovium of patients with RA and is considered to be functionally important since treatment with an anti-MMP14 antibody was found to inhibit cartilage destruction in arthritic mice [88,90]. MMP-3 is known to be a valid marker of the disease and a predictor of progressive cartilage destruction [91]. Hence, the levels of MMP-3 might reflect the activity of tissue-destructive synovial fibroblasts.

The most significant difference between RA and OA, therefore, is the cellular source of cartilage-destructive enzymes involved. In OA, chondrocytes primarily produce MMPs and ADAMTS families, whereas, in RA, activated synovial fibroblasts damage the joints. However, RA and OA likely share a common mechanism for cartilage destruction, in part, since inflammation has been suggested to participate in the etiology of OA. For instance, gremlin-1, an antagonist of bone-forming proteins induced by mechanical loading, promotes the production of MMPs and ADANTS-5 and inhibits type 2 collagen and aggrecan production in OA [92]. Similarly, the expression of gremlin-1 is elevated in RA synovium [93]. Although OA is a degenerative disease and RA is an autoimmune disease, some of the mechanisms (cartilage degeneration due to inflammation) are similar. Thus, studies on OA can immensely contribute to a better understanding of the pathogenesis of cartilage destruction in RA.

## 5. Fibroblasts as the Critical Mediators of Joint Destruction in RA

Fibroblasts have been recognized as scaffold cells for living organisms. However, recent developments in analytical technologies have revealed that diverse subsets of fibroblasts, including pro-inflammatory and tissue-destructive types, exist in diseased tissues, such as in RA [8,9,10,18,19,20], enteritis, and cancer, and that they are closely associated with pathogenesis, interacting with the surrounding cells [94,95]. Here, we have discussed the crosstalk between synovial fibroblasts and immune cells, as well as the phenotype and heterogeneity of fibroblasts that intervene in inflammation and joint destruction in RA.

### 5.1. Interaction between Synovial Fibroblasts and Immune Cells

**Figure 3:**
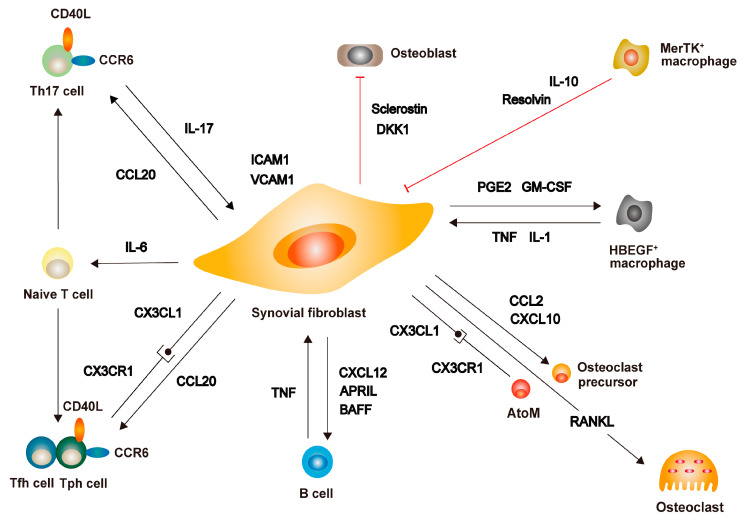
Cross-talk of synovial fibroblasts and immune cells. Synovial fibroblasts interact with a variety of immune cells. T cells expressing CD40L upregulate the expression of intercellular adhesion molecule 1 (ICAM1) and vascular cell adhesion molecule 1 (VCAM1), to promote the production of interleukin-6 (IL-6) by synovial fibroblasts. IL-6 produced by synovial fibroblasts, in turn, promotes the differentiation of T helper 17 (Th17) cells producing IL-17 and T follicular helper (Tfh)/T peripheral helper (Tph) cells producing autoantibodies. Chemokine (C-C motif) ligand 20 (CCL20) and chemokine (C-X3-C motif) ligand 1 (CX3CL1) from synovial fibroblasts recruit chemokine (C-C motif) receptor 6 (CCR6)- and chemokine (C-X3-C motif) receptor 1 (CX3CR1)- positive T cells to the inflamed synovium. The tumor necrosis factor (TNF) from B cells stimulates synovial fibroblasts, chemokine (C-X-C motif) ligand 12 (CXCL12) from synovial fibroblasts supports B cell survival, and TNF ligand superfamily members 13 and 13B (APRIL and BAFF) from synovial fibroblasts promote the differentiation and activation of B cells. Monocyte lineage cells are recruited to the inflamed synovium by the action of CCL2 and CXCL10 produced by synovial fibroblasts and differentiate into osteoclasts through the action of receptor activator of NF-κB ligand (RANKL). Recently identified arthritis-associated osteoclastogenic macrophages (AtoMs) interact with synovial fibroblasts through the CX3CL1/CX3CR1 axis. Prostaglandin E2 (PGE2) and granulocyte-macrophage colony-stimulating factor (GM-CSF) secreted by synovial fibroblasts induce the generation of pro-heparin-binding EGF-like growth factor (HBEGF)-positive macrophages, while TNF and IL-1 produced by these HBEGF-positive macrophages stimulate synovial fibroblasts. Conversely, MerTK-positive macrophages exert suppressive effects on synovial fibroblasts through IL-10 and Resolvin. Furthermore, Dickkopf-related protein 1 (DKK1) and sclerostin from synovial fibroblasts suppress osteoblast differentiation.

Synovial fibroblasts cooperate with immune cells to destroy joints in RA (Figure 3).

T cells expressing CD40L act on synovial fibroblasts to promote the expression of intercellular adhesion molecule 1 (ICAM1) and vascular cell adhesion molecule 1 (VCAM1) in addition to IL-6 [96]. Conversely, ICAM1 and VCAM1 produced by synovial fibroblasts activate CD4+ T cells and solidify the consolidation of T cells with synovial fibroblasts [97,98]. T cells accelerate the expression of IL-6 and IL-8 by synovial fibroblasts [99], and IL-7 expressed by synovial fibroblasts proliferates T cells [33]. Chemokine (C-X3-C motif) ligand 1 (CX3CL1, fractalkine)-expressing synovial fibroblasts recruit chemokine (C-X3-C motif) receptor 1 (CX3CR1)-positive T cells [100]. In addition, CCL20 from synovial fibroblasts recruits chemokine (C-C motif) receptor 6 (CCR6)-expressing T cells [101]. The high levels of expression of CX3CR1 and CCR6 in recently identified Tph cells suggested the interplay between synovial fibroblasts and T cells in the inflamed synovium [23]. In addition, it is of a matter of importance that sub-lining synovial fibroblasts secrete IL-6 to promote Th17 cell differentiation, and IL-17 further stimulates IL-6 production from sub-lining synovial fibroblasts and synovial macrophages forming a positive feedback loop. Additionally, Th17 cells facilitate RANKL production from the lining synovial fibroblasts [21,22,38,39]. Considering the presence of a THY1+HLAhi population in the sub-lining synovial fibroblasts [18], the latter may be considered to function as APC for sustaining inflammation.

Focusing on the interactions with B cells, synovial fibroblasts were found to support B cell survival via the expression of VCAM1 and CXCL12 [102]. Additionally, synovial fibroblasts stimulated by Toll-like receptor 3 ligands produced TNF ligand superfamily members 13 and 13B (APRIL and BAFF) to promote B cell differentiation and activation [103]. In a recent study, it was reported that, prior to a flare of RA, B cell activation was followed by the expansion of circulating CD3-CD45-PDPN+ pathogenic sub-lining fibroblast-like pre-inflammatory mesenchymal (PRIME) cells [104]. This suggested the presence of an interplay of B cells with synovial fibroblasts during the relapse of RA.

Synovial fibroblasts guide monocyte lineages to inflamed synovium via CCL2 and CXCL10 [105]. Normally, osteoclasts contribute to bone remodeling on the bone surface; however, in RA, osteoclasts are present within the proliferating synovium. Osteoclasts localized at the junction region between the pannus and bone were not known to have been derived from the same progenitor cells as osteoclasts in the bone marrow. In this regard, a characteristic macrophage population of CX3CR1hiLy6CintF4/80+I-A/I-E+ migrating from peripheral blood was reported as one of the osteoclast precursors, and this population was named arthritis-associated osteoclastogenic macrophages (AtoMs) [106]. Since synovial fibroblasts in RA express CX3CL1, the AtoMs-synovial fibroblast axis may be crucial in inflammatory joints. Furthermore, prostaglandins produced by synovial fibroblasts induce pro-heparin-binding EGF-like growth factor (HBEGF)-positive macrophages [107]. In turn, HBEGF+ macrophages increase the aggressiveness of synovial fibroblasts. In contrast, there are reports on the macrophage population that have an inhibitory role in inflammation. CX3CR1+ synovial macrophages form a tight-junction-mediated barrier to protect the joint from inflammation [108]. The synovium of patients with RA, who are able to maintain remission with therapy, is abundant in MerTK+CD206+ macrophages that ameliorate inflammation and induce repair capacity of synovial fibroblasts by the lipid mediators [109].

As discussed earlier in this article, synovial fibroblasts produce RANKL to promote osteoclast formation and inhibit osteoblast formation under inflammatory conditions at the same time [21,22,58]. Synovial fibroblasts in RA suppress osteoblast differentiation via DKK1 and sclerostin [26,27]. In addition, pro-inflammatory cytokines produced by immune cells inhibit osteoblast differentiation [21,22,58].

Thus, recent developments in analytical techniques have gradually elucidated the existence of subsets with different roles in each cell population and their interactions.

### 5.2. Phenotype and Heterogeneity of Synovial Fibroblasts in RA

Synovial fibroblasts are highly activated in RA and have proliferative and invasive properties [110]. At the onset of RA, the DNA methylation pattern of synovial fibroblasts already differs from that of healthy individuals, suggesting that the epigenetic alteration could be a cause of disease initiation rather than an effect of arthritic conditions [111]. Synovial fibroblasts of patients with RA also undergo metabolic changes. Hypoxic condition is regarded as a characteristic of the RA synovium microenvironment. Glycolysis, which produces ATP under hypoxic conditions, increases in the synovial fibroblasts in RA [110]. The expression of hypoxia-inducible factor 1α (HIF1α) is linked with an aggressive phenotype of synovial fibroblasts [112]. In addition, an important surface marker of RA-related synovial fibroblasts is cadherin-11 [113]. Cadherin-11 functions as an activator of MAPK and NF-κB, stimulating synovial fibroblasts to produce IL-6 [114].

As mentioned above, the heterogeneity of synovial fibroblasts has attracted much attention in recent years. RA-related synovial fibroblasts have phenotypically and functionally distinct cell subsets, namely the pro-inflammatory subset in the sub-lining layer and the tissue-destructive subset in the lining layer [8,9,10,18,19,20]. scRNA-seq analysis has divided RA-related synovial fibroblasts, which are characterized by podoplanin (PDPN) expression, into three subpopulations, namely CD34-THY1-, CD34-THY1+, and CD34+ groups. Among them, CD34-THY1+ cells accumulate around the perivascular areas in the synovium tissue and produce pro-inflammatory cytokines with a high proliferative capacity [9]. A combination of new technologies, namely scRNA-seq and mass cytometry, showed that CD34-THY1+HLA-DRhi synovial fibroblasts are increased and support IL-6 and CXCL12 production [18]. Additionally, another scRNA-seq analysis focused on RA synovial fibroblasts that express fibroblast activation protein-α (FAPα) as the pathogenic cell subset. FAPα+THY1+ synovial fibroblasts, localized in the sub-lining lesion, displayed inflammatory phenotype while FAPα+THY1- synovial fibroblasts, localized in the lining lesion, displayed tissue-destructive phenotype. It would be worth noting that the adoptive transfer of FAPα+THY1+ cells induced inflammation while FAPα+THY1- cells induced joint destruction in the mouse model of serum transfer-induced arthritis [8]. The polarization of synovial fibroblasts toward pro-inflammatory and tissue-destructive properties is a very important issue. The binding of Notch ligands of endothelial cells to NOTCH3 on sub-lining synovial fibroblasts stimulates Notch signaling, resulting in the differentiation of THY1+ synovial fibroblasts that produce pro-inflammatory cytokines [20]. Inhibition of NOTCH3 signaling by antibody or gene knockout of *Notch3* suppressed inflammation and bone destruction in the mouse model of serum transfer-induced arthritis. Furthermore, epigenomic analysis of RA synovium provided novel insights into tissue-destructive fibroblasts. The most important feature of tissue-destructive fibroblasts is RANKL production, and a synovial fibroblasts-specific RANKL enhancer E3 has been identified. Even under arthritic conditions, RANKL expression in synovial fibroblasts was reduced, and bone destruction was ameliorated, in E3-deficient mice [10]. Moreover, ETS1, the transcriptional factor that binds to E3, has been reported to induce not only RANKL but also cartilage-degenerative factors, such as MMP-3 and MMP-13 [10]. Both bone and cartilage destruction were suppressed in arthritic mice lacking ETS1 in synovial fibroblasts, although the degree of inflammation remained unchanged [10].

Overall, the heterogeneity of synovial cells, especially synovial fibroblasts, in RA, and the immune response mediated by fibroblasts are gradually becoming clear. The interaction between synovial fibroblasts and immune cells plays a very critical role in joint destruction in RA, and a new field that could be called stromal immunology is steadily developing. Understanding the pathogenesis based on various functions possessed by fibroblasts could lead to the development of novel therapies for diseases such as enteritis and cancer, as well as RA, in the future.

## 6. Conclusions

Studies on bone destruction in RA have revealed bone-immune interactions at molecular levels. Recently, attention has also been directed toward periarticular and systemic bone loss in RA, both of which have different mechanisms. In the context of bone destruction in inflamed joints, new analytical techniques, led by scRNA-seq, have provided insight into the heterogeneity of synovial cells that express the key factors. As discussed in this review, synovial fibroblasts are the primary contributors to joint destruction in RA and represent a promising therapeutic target. However, according to the fibroblast atlas based on single-cell analysis data, there are only two universal fibroblast transcriptional subtypes in mice, which are highly similar to those in humans [115]. In other words, immediate targeting of synovial fibroblasts in RA as a therapeutic strategy raises concerns about the potential side effects such as impaired wound healing. Regarding the structural damage in RA, cartilage destruction is a crucial aspect, but its mechanism is not associated with RANKL. Furthermore, clinical trials targeting the inhibition of MMPs, one of the causes of cartilage destruction, have failed due to their toxicity to the musculoskeletal system [116]. Based on these factors, the most effective treatment for RA at present is therapy targeting the immune system. Although patients with RA exhibit high heterogeneity, effective stratification of subgroups may lead to better personalized treatment choices and potentially reduce the number of patients classified as D2T. Moreover, combining single-cell transcriptomics with techniques such as mass cytometry can lead to the identification of novel and more detailed subsets of pathological synovial fibroblasts in RA, which, in turn, can contribute to the development of more personalized and novel therapies.

## Data Availability

Not applicable.
